# Transgenic *Drosophila melanogaster* Carrying a Human Full-Length DISC1 Construct (UAS-*hflDISC1*) Showing Effects on Social Interaction Networks

**DOI:** 10.3390/cimb46080502

**Published:** 2024-08-03

**Authors:** Bobana Samardžija, Milan Petrović, Beti Zaharija, Marta Medija, Ana Meštrović, Nicholas J. Bradshaw, Ana Filošević Vujnović, Rozi Andretić Waldowski

**Affiliations:** 1 Faculty of Biotechnology and Drug Development, University of Rijeka, Radmile Matejčić 2, 51 000 Rijeka, Croatia; bobana.samardzija@biotech.uniri.hr (B.S.); beti.zaharija@biotech.uniri.hr (B.Z.); marta.medija@student.uniri.hr (M.M.); nicholas.b@biotech.uniri.hr (N.J.B.); 2 Faculty of Informatics and Digital Technologies, University of Rijeka, Radmile Matejčić 2, 51 000 Rijeka, Croatia; milan.petrovic@inf.uniri.hr (M.P.); amestrovic@inf.uniri.hr (A.M.)

**Keywords:** human full-length *Disrupted in Schizophrenia 1* (*hfl-DISC1*), *Drosophila melanogaster*, social interaction networks (SINs), locomotor activity, redox regulation

## Abstract

Disrupted in Schizophrenia 1 (DISC1) is a scaffold protein implicated in major mental illnesses including schizophrenia, with a significant negative impact on social life. To investigate if DISC1 affects social interactions in *Drosophila melanogaster*, we created transgenic flies with second or third chromosome insertions of the human full-length *DISC1* (*hflDISC1)* gene fused to a UAS promotor (UAS-*hflDISC1*). Initial characterization of the insertion lines showed unexpected endogenous expression of the DISC1 protein that led to various behavioral and neurochemical phenotypes. Social interaction network (SIN) analysis showed altered social dynamics and organizational structures. This was in agreement with the altered levels of the locomotor activity of individual flies monitored for 24 h. Together with a decreased ability to climb vertical surfaces, the observed phenotypes indicate altered motor functions that could be due to a change in the function of the motor neurons and/or central brain. The changes in social behavior and motor function suggest that the inserted *hflDISC1* gene influences nervous system functioning that parallels symptoms of DISC1-related mental diseases in humans. Furthermore, neurochemical analyses of transgenic lines revealed increased levels of hydrogen peroxide and decreased levels of glutathione, indicating an impact of DISC1 on the dynamics of redox regulation, similar to that reported in transgenic mammals. Future studies are needed to address the localization of DISC1 expression and to address how the redox parameter changes correlate with the observed behavioral changes.

## 1. Introduction

**Figure 1:**
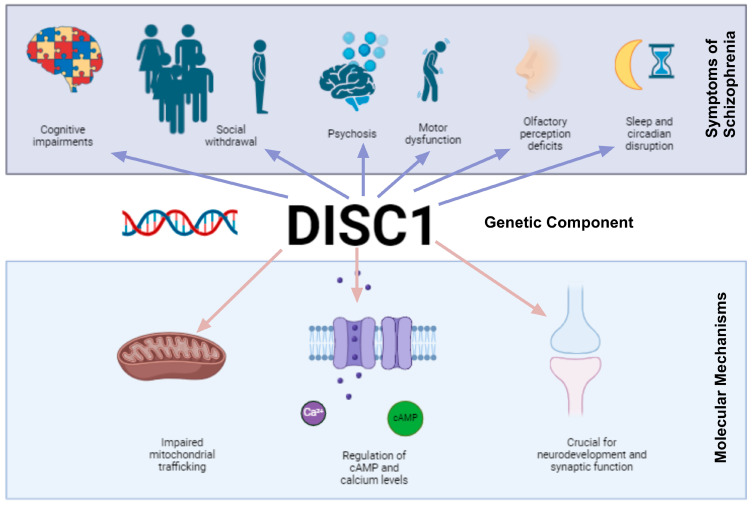
DISC1 is a critical scaffold protein involved in multiple molecular mechanisms that affect neurodevelopment and synaptic function. Its dysregulation is linked to several symptoms of schizophrenia, including cognitive impairments, social withdrawal, psychosis, olfactory perception deficits, motor dysfunction, and disruptions in sleep and circadian patterns.

Scaffold proteins in neurons form complexes with multiple proteins required for the organization of the postsynaptic signaling machinery [1]. They are involved in receptor localization, axonal branching, and neuronal communication at the postsynaptic terminals ultimately, influencing neurotransmission [2]. An important scaffolding protein involved in both neurodevelopment and dopamine signaling is Disrupted in Schizophrenia 1 (DISC1) [3,4]. The *DISC1* gene was originally identified in a large family, where it was strongly linked to the presence of major mental illnesses, including schizophrenia (SCZ) [5,6,7]. More recently, insoluble aggregates of DISC1 have been confirmed in the brain and cerebral spinal fluid of patients with SCZ [8,9]. The behavioral symptoms of SCZ include social withdrawal [10], cognitive impairments [11], and psychosis [12] along with less known deficits in olfactory perception [13], motor dysfunction [14], and disruptions in circadian [15] and sleep patterns [16]. The factors contributing to the onset and progression of these symptoms are not fully understood, although SCZ has a strong genetic component (Figure 1).

As a scaffold protein, DISC1 interacts with a wide array of proteins, crucial for various aspects of neurodevelopment and synaptic function [4]. Interaction partners are involved in functions including the regulation of cyclic adenosine monophosphate and calcium levels in the cell, important for mitochondrial trafficking by molecular motors using the motor–adaptor complex [17,18]. It was reported that mutations in DISC1 impair mitochondrial trafficking, and that the reduction in DISC1 function leads to mitochondrial dysfunction [19,20,21,22]. This is particularly important since reactive oxygen species (ROS) have been reported to be associated with neurodegenerative disease and other neurological disorders, such as SCZ [23,24,25]. Nuclear magnetic resonance studies conducted on humans have shown a correlation between glutathione (GSH) antioxidant status and SCZ [26,27,28,29]. It is therefore possible that this is caused, at least in part, by disrupted mitochondrial trafficking due to mutation or dysregulation of DISC1, and possibly mitochondria GSH cascades that underlie non-enzymatic regulation of redox status in the cells.

Although replicating human psychiatric symptoms in animal models, especially in species as evolutionarily distant as *Drosophila*, is challenging, recent research frameworks for mental disorders emphasize the importance of understanding core underlying mechanisms of brain circuit dysfunction which are conserved across species [30]. In line with this concept, several studies have used fly models in recent years to investigate the molecular and genetic mechanisms of various mental disorders [31,32,33,34,35,36]. Genetically modified animal models can be used to explore the molecular, physiological, and behavioral mechanisms underlying the development and progression of SCZ. Many DISC1 transgenic models have been generated, leading to a variety of behavioral phenotypes [37]. 

A homolog of the *DISC1* gene is not present in the fly genome; however, *Drosophila* has counterparts for more than 90% of established DISC1 interacting proteins [38,39]. For example, a C-terminal truncated mutant DISC1 downregulates three pathways involving the *Drosophila* genes *Dystrophin*, *Trio*, and *Shot* [40]. DISC1 overexpression in the mushroom body neurons of the larval brain impair associative olfactory memory due to axonal and dendritic branching defects. The same study also pinpointed the amino-terminal domain of DISC1 as crucial for memory suppression [39]. Adult transgenic flies expressing human full-length DISC1 in the mushroom body show sleep disturbances connected to CREB signaling and gene transcription regulation [32]. Expression of full-length or truncated forms of DISC1 in the glutamatergic neurons of the neuromuscular junction in *Drosophila* showed that DISC1 regulates the development of glutamatergic synapses of the motor neurons [38]. The similarity between the cellular and behavioral phenotypes induced by DISC1 expression in flies and those in humans argue that *Drosophila* is an excellent model organism for describing the mechanisms of DISC1 action.

In recent decades, *Drosophila* has become a valuable model for the study of social behavior using the analysis of social interaction networks (SINs). This is especially relevant for the modeling of SCZ symptoms considering the impact that SCZ has on social interactions in humans. We and others have recently demonstrated that *Drosophila* provides an excellent model system for analyzing social behavior using the analysis of SINs [41]. SIN analysis describes the pattern and the structure of interactions between individuals from a video recording of a group of flies in an open recording arena. Based on the frequency or duration of interactions, it is possible to quantify the strength of social bonds. For example, the measure of the degree centrality describes the number of connections that each fly has, indicating its level of social activity, while the clustering coefficient defines the tendency of flies to form tightly knit groups [42,43,44]. 

In *Drosophila*, an important genetic tool for mechanistic and neural network analysis is the binary expression system UAS/GAL4 that allows for precise spatial and temporal gene expression [45]. Our initial aim was to overexpress the DISC1 protein (the UAS-*hflDISC1* construct) after activation with a spatially regulated expression of the GAL4 transcription factor which binds the UAS sequence. The overexpression of *hflDISC1* in rats leads to the aggregation of the DISC1 protein and behavioral changes [46], which reflect similar aggregates seen in human patients with SCZ and other major mental illnesses [8,47].

First, to ensure that the genomic integration of the UAS-*hflDISC1* construct did not cause unintended gene disruption at the insertion site, nor lead to DISC1 expression due to endogenous activation, we characterize here the behavioral and the neurochemical phenotypes of the two lines that we created, the second and third chromosome insertions, UAS-*hflDISC1-2nd* and UAS-*hflDISC1-3rd* [48,49]. We then use SIN analysis to characterize the social behavior among a group of flies, with the locomotor activity and sleep for 24 h as indicators of spontaneous behavior in individual flies and the negative geotaxis, a startle-induced reflexive behavior evident as the ability to climb the vertical surfaces. To confirm that there is no activation of the UAS-*hflDISC1* construct, we quantify the DISC1 protein in the headless bodies and the heads of transgenic flies. Finally, to determine if the transgene led to the perturbation of the redox regulation, we measure hydrogen peroxide as a reactive oxygen species (ROS) that can cause cellular damage and glutathione (GSH), a major antioxidant that helps neutralize ROS.

## 2. Materials and Methods

### 2.1. Fly Lines

A plasmid encoding human full-length *DISC1* (*hflDISC1*) was purchased from the DNASU Plasmid Repository (clone HsCD00516321, Arizona State University, Tempe, AZ, USA). LR clonase II (Thermo Fisher Scientific, Waltham, MA, USA) was then used to transfer the reading frame to the *pPRW* vector (RRID:DGRC_1137, Indiana University, Bloomington, IN, USA, supported by NIH Grant 2P40OD010949). As part of P-element transgenesis, the *pPRW-hflDISC1* vector and a P element helper plasmid were microinjected into white *w^1118^* embryos (service by the Department of Genetics, University of Cambridge, UK). Transformant chromosomes were balanced by using *SM6a* (2nd chromosome balancer) on flies *w[1118][iso]/y[+]Y; Sco/SM6a; 3[iso]* and *TM6C* (3rd chromosome balancer) on flies *w[1118][iso]/y[+]Y; 2[iso]; TM2/TM6C*, *Sb*. Hemizygote flies carrying transgenic construct UAS (promotor) fused with the *hflDISC1* gene, balanced on the 2nd chromosome (UAS-*hflDISC1-2nd*) or 3rd chromosome (UAS-*hflDISC1-3rd*), were generated. As control groups, we used the white *w^1118^* line from Bloomington Stock Center (5905).

### 2.2. Fly Husbandry

Flies were grown on standard cornmeal media, with the addition of the anti-fungal agent methyl parahydroxybenzoate (≥98% purity, Carl Roth, Karlsruhe, Germany) and propionic acid (≥99.5% purity, Merck, Darmstadt, Germany) at 25 °C, 70% humidity, and on a 12 h dark–light cycle (lights on at 08:00, lights off at 20:00).

### 2.3. Behavioral Assays

#### 2.3.1. Social Interaction Network Analysis

The 3–5-day-old male flies of each genotype, collected using CO_2_ anesthesia one day earlier, were transferred into groups of 12 flies in a circular arena (61 mm in diameter). After 10 min of adaptation, a video was recorded for 25 min starting at 10:00, as described previously [41]. FlyTracker software, https://github.com/kristinbranson/FlyTracker (accessed on 20 April 2022) running on the MATLAB platform, was used to assign the identity, spatial position, and orientation of each fly. This information was used to define social interaction networks (SINs), in which flies had to be within a 2.5 body length distance of each other (approximately 5 mm) and facing each other (within a 160 degree angle for a minimum of 0.6 s), as standardized previously [42,50]. A Python script was used to analyze the SIN parameters, as described previously [51]. These SINs were weighted in directed graphs containing two sets of data: nodes (individual flies) and edges (interactions between two flies). Directed edges in this approach are used to address the direction of the relationship. For example, an arrow pointing from A to B signifies that fly A interacted with fly B. In the network analysis, two types of weights were used: number of interactions (count) and total duration of interactions (duration). SIN parameters were analyzed at both global and local levels, with the global level analyzing the properties of the entire network and local level analyzing connections and behavior of an individual fly within the network. The parameters were global efficiency, local level clustering coefficient, betweenness centrality [52], and closeness centrality [41].

All SIN data were normalized using z-scores generated by comparing real (observed) and random networks, according to the formula (measurement_real_ − mean(measurement_random_)/standard deviation (measurement_random_) [44]. For each experimental group, 10,000 random networks were calculated by selecting 12 random flies from different measurements of the same experimental group and analyzing their data as if they were a single group.

#### 2.3.2. Monitoring of the Locomotor Activity

Locomotor activity was measured using a Drosophila Activity Monitoring System (DAMS, TriKinetics, Waltham, MA, USA) with one infrared beam. Sixteen adult male flies, 3–5 days old or 27–30 days old, from each genotype were transferred by aspiration into 65 mm × 5 mm glass tubes with cornmeal media and a porous plug, each on separate end of tube. Flies were tracked for five consecutive days at 25 °C, under a 12 h light/12 h dark cycle. Activity was defined as the number of times that a fly broke the beam by crossing the middle of the glass tube in one-minute intervals. Locomotor activity data collected using DAMSystem3 Data Collection Software https://trikinetics.com/ (accessed on 20 April 2022.) were exported as .txt files using the FileScan program (www.trikinetics.com) and were analyzed with software developed in our laboratory [53]. Activity was expressed as the average number of beam breaks per minute during an hour (for the line graphs) or during the 12 h for the activity during the light on or light off periods.

#### 2.3.3. Negative Geotaxis

The negative geotaxis test defines the ability of flies to climb a vertical surface after being dropped to the bottom of the recording vial [54]. Testing was performed on males 3–5 days old separated one day before testing into culture vials with food. On the day of the test, 10 flies were placed in vials without food for 30 min for adaptation. The vials are then struck three times onto a surface for all the flies to fall at the bottom of the vial, and then photographed 5 s later, to determine the percentage of flies that have climbed 5 cm height or over. Measurements were repeated five times for each group, with an interval of one minute between measurements.

### 2.4. Biochemical Assays

#### 2.4.1. Western Blot Analysis

Homogenates were generated from 5 headless bodies or 20 heads per genotype. Samples were lysed on ice for 10 min using RIPA buffer (50 mM Tris-HCl, 150 mM sodium chloride, 0.1% NP-40, 0.5% sodium deoxycholate 0.1% SDS, pH 8) with the addition of cOmplete^TM^, EDTA-free Protease Inhibitor Cocktail (Roche, Basel, Switzerland), followed by centrifugation at 10,000 rpm for 45 min at 4 °C. For protein precipitation, cold acetone was added to the supernatant and incubated at 4 °C, overnight. Protein concentration was determined on a BioDrop Touch Duo, BioDrop (serial number: BD1607). After precipitation, acetone was removed, and proteins were denatured in 156 mM Tris pH 6.8/5% SDS/20 mM DTT/25% glycerol with bromophenol blue at 95 °C for 4 min. Proteins were than separated on bis-acrylamide gels and transferred to PVDF membranes (Macherey-Nagel, Düren, Germany) using a Transblot Turbo system (Bio-Rad, Hercules, CA, USA). Confirmation of protein transfer was conducted using 0.5% Ponceau S/2% acetic acid. Membranes were blocked at room temperature for 45 min in PBS-T (phosphate buffered saline/0.05% Tween-20) containing 5% milk powder, followed by staining with primary antibodies (anti-DISC1, 40-6800, Thermo Fisher Scientific, 1000-fold dilution or anti-actin, A3854, Merck, 10,000-fold dilution) diluted in the same buffer overnight at 4 °C. Membranes were washed with PBS-T for 30 min. Staining with a secondary antibody (Pierce Goat Anti-Mouse IgG (H+L) Peroxidase Conjugated 31430 or Pierce Goat anti-Rabbit IgG (H+L), Peroxidase Conjugated 31460, both 10,000-fold dilution in PBS-T) was conducted for one hour at room temperature, as described above. Signals were detected using a ChemiDoc MP Imaging System accompanied by ImageLab 5.2 software (Bio-Rad), after visualization with ECL (Thermo Fisher Scientific).

#### 2.4.2. Hydrogen Peroxide Concentration Measurement

Samples of 5 headless bodies and 32 head homogenates were prepared as previously described [55]. Briefly, after mechanical homogenization, samples were incubated for 30 min at 37 °C in the dark and then the H_2_O_2_ concentration was measured indirectly using dihydroethidium (DHE) and a microplate reader (Infinite 200PRO, Tecan, Männedorf, Switzerland) with an excitation wavelength of 480 nm and emission at 625 nm.

#### 2.4.3. Glutathione (GSH/GSSH) Concentration Measurement

Samples of 5 headless bodies and 32 heads were treated with PBS containing 5% trichloroacetic acid (TCA) as extraction buffer (10 min, in ice bath). After homogenization and centrifugation at 14,000 rpm and 4 °C, the supernatant was collected. Ellman’s method was used to measure reduced, oxidized, and total glutathione (GSH) [56]. For measurements of free GSH (reduced form), we used 110 μL of R1 solution (10.0 mM EDTA in TRIS (500 mM, pH 8.2)), 10 μL of R2 (10.0 mM 5,5′-dithiobis(2-nitrobenzoic acid) (DTNB) in methanol), and 10 μL of sample. After mixing all solutions in a 48 well plate, we measured the absorbance of the produced 2-nitro-5-thiobenzoate anion (TNB^2−^) ions at 415 nm using an microplate reader (Infinite 200PRO, Tecan, Männedorf, Switzerland) To determine the concentration of GSSG (the oxidized form), we reduced samples to GSH using RR (10 mL fresh, 3.5 M NaBH_4_, 1.5 M NaOH in a 1:1 mix of water–methanol). In 60 μL of a sample after homogenization, we added 15 μL of RR and waited for 15 min, after which we added 7 μL of concentrated HCl. Total GSH was measured using 110 μL of R1, 10 μL of R2, and 10 μL of reduced sample. Free GSH was measured directly after homogenization, and the total GSH concentration measured after the reduction of GSSG to GSH in the samples was determined using a calibration curve of known GSH concentrations. To determine GSSH concentration, we subtracted free GSH from total GSH and divided by two.

### 2.5. Data Analysis and Statistics

All statistical analyses and visualizations were performed using Prism (version 10.2.3, GraphPad, La Jolla, CA, USA). All data were tested for normality using Bartlett’s test or Brown–Forsythe’s test. Differences between groups were analyzed using one-way ANOVA or Kruskal–Wallis ANOVA, followed by Tukey’s multiple comparisons test, as appropriate for the data set. Differences were considered significant if *p* < 0.05.

## 3. Results

### 3.1. Reduced Social Dynamics and Organizational Structure in Flies with hflDISC1

Considering that DISC1 is associated with the development of mental illnesses and the impairment of interpersonal relationships, we investigated if social interaction phenotypes are affected in transgenic flies carrying an insertion of *hflDISC1*, either on the second chromosome (UAS-*hflDISC1*-*2nd*) or the third (UAS-*hflDISC1*-*3rd*). Social interaction network (SIN) analysis was performed using video recordings of 20 flies freely moving in an open field arena, with a minimum of 15 replicates for each genotype.

**Figure 2:**
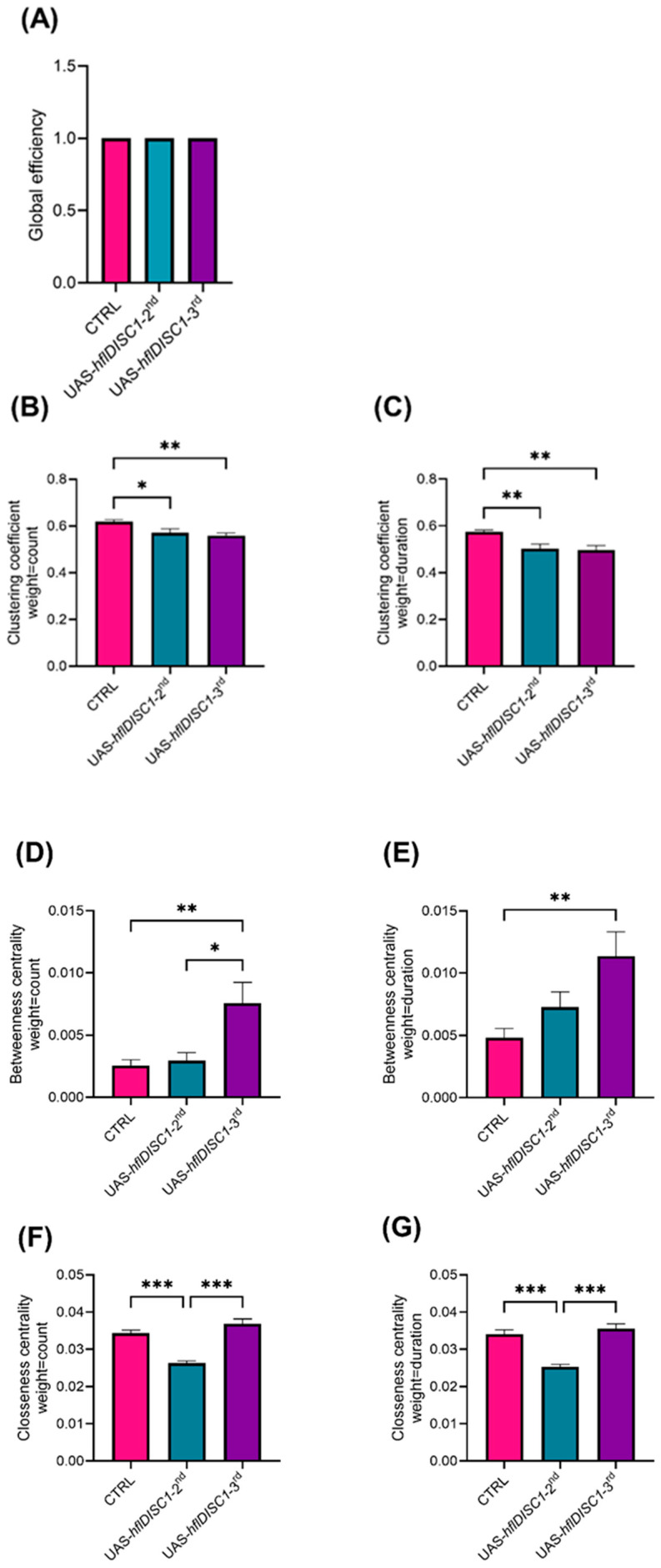
Centrality measures of social interaction networks in UAS-*hflDISC1* flies depend on site of insertion**.** All measurements were performed using n > 15 replicates of UAS*-hflDISC1-2nd* and UAS-*hflDISC1*-*3rd* and control *w^1118^* male flies 3–5 days old. The parameters: global efficiency (**A**) and clustering coefficient (**B**,**C**), weighted either by the count (number) (**B**) or the duration (seconds) (**C**), were statistically analyzed using one-way ANOVA with Tukey’s multiple comparison post hoc test. Parameters betweenness centrality (**D**,**E**) and closeness centrality (**F**,**G**), weighted either by the count (number) (**D**,**F**) or the duration (duration) (**E**,**G**), were statistically analyzed using Kruskal–Wallis with Dunn’s test. *p* value style: APA < 0.05 (*), <0.01 (**), <0.001 (***).

We observed high global efficiency for both UAS-*hflDISC1* lines as well as for *w^1118^* controls, which indicates a well-connected network in which interactions between flies in a SIN spread quickly and easily, as would be expected for highly social and cooperative groups of flies (Figure 2A). This suggests that the genomic insertion of the UAS-*hflDISC1* does not fundamentally impair social interaction and that efficient communication within the SIN still occurs.

To assess local connectivity and social cohesion within SINs, we analyzed the clustering coefficient of the SINs. Flies with the UAS-*hflDISC1* insertion exhibited smaller clustering coefficients compared to controls, suggesting lower densities of interactions with distinct group formation tendencies (Figure 2B,C). This effect was consistent regardless of the duration or frequency of interaction or of the insertion site. Analysis of betweenness centrality revealed notable differences between UAS-*hflDISC1*-*2nd* and UAS-*hflDISC1*-*3rd* lines. UAS-*hflDISC1*-*3rd* flies exhibited higher betweenness centrality, indicating more individuals playing critical connector roles in the network with enhanced social cohesion (Figure 2D,E). In contrast, the closeness centrality analysis of UAS-*hflDISC1*-*2nd* flies was lower, suggesting a more fragmented network structure (Figure 2F,G). UAS-*hflDISC1*-*3rd* flies exhibited closeness centrality the same as the control, indicating greater efficiency in spreading information or interacting within the network.

Overall, significant differences in SIN parameters were observed between UAS-*hflDISC1* lines inserted on the second versus the third chromosome, particularly in terms of betweenness and closeness centrality. These findings suggest distinct impacts of an *hflDISC1* transgenic construct on the social dynamics and organizational structure of *Drosophila* communities.

### 3.2. Altered Locomotor Activity and Sleep Patterns in the UAS-hflDISC1 Transgenic Flies

Locomotor activity influences SINs by affecting the frequency, timing, and nature of interactions among flies. Flies that have inherently higher locomotor activity will encounter and interact with more individuals, shaping the structure and dynamics of the network. It is therefore possible that the observed changes in SIN parameters are related to a change in the amount of locomotor activity. To investigate this, we measured the average amount of locomotor activity of individual flies during 24 h for five days with a one-minute resolution. To additionally explore whether aging influences the intensity of the UAS-*hflDISC1* phenotype, we assessed locomotor activity in 3–5- and 27–30-day-old male flies.

**Figure 3:**
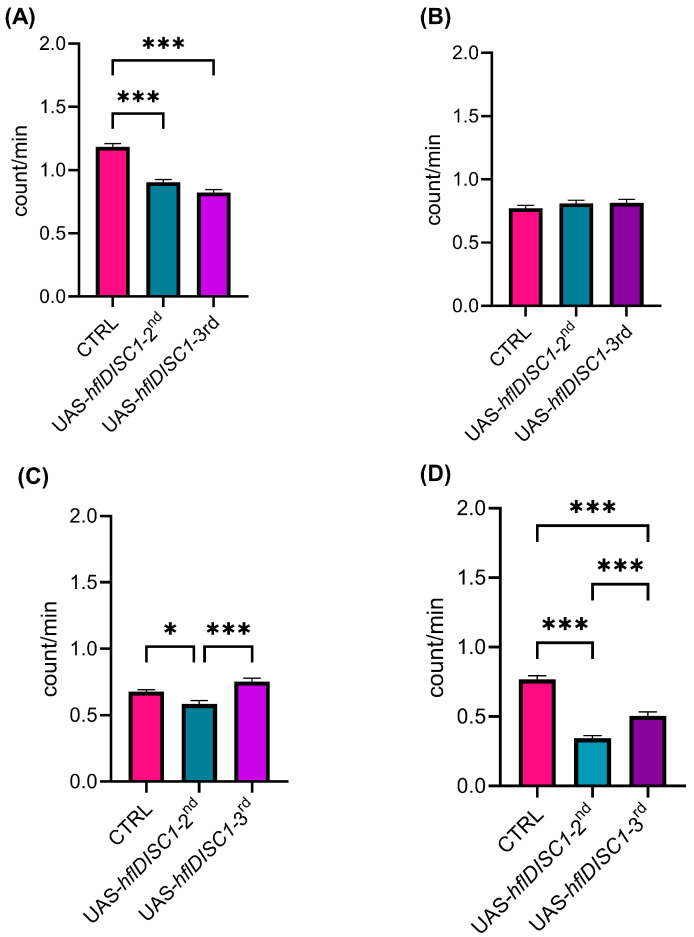
The change in the locomotor activity in UAS-*hflDISC1* transgenic flies is age-dependent. (**A**,**B**), young flies 3–5 days old, and (**C**,**D**), flies aged 27–30 days. Activity was measured as the average value of the number of crossings of the middle of the tube in 1 min resolution from 16 individual flies per genotype. Data are then plotted as the average number of crossings of the midline of the tube averaged for 12 h (12 h light–12 h dark) for five days. Average day time activity ± SEM from 08:00 to 20:00 (**A**,**B**), and average activity during the night ± SEM from 20:00 to 08:00 (**C**,**D**). One-way ANOVA with Tukey’s multiple comparison post hoc test. *p* value style: APA < 0.05 (*) and <0.001 (***).

**Figure A1:**
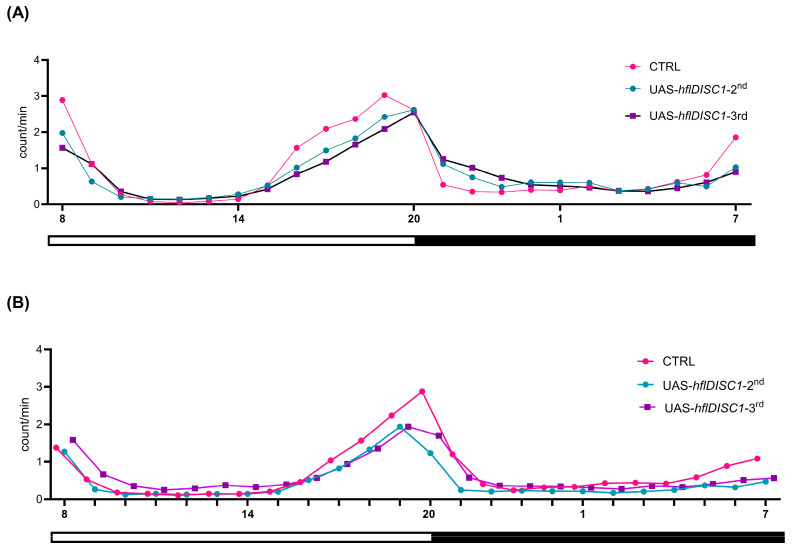
The change in the locomotor activity in UAS-*hflDISC1* transgenic flies is age-dependent. (**A**), young flies 3–5 days old, and (**B**) flies aged 27–30 days. Activity was measured as the average value of the number of crossings of the middle of the tube in 1 min resolution from 16 flies per genotype. Data were then plotted as activity per one hour (average of 60 min interval from 16 flies). Line graphs are an average of 5 consecutive days of activity measurement in the DAMS system presented hour by hour.

The 24 h locomotor activity of flies has two distinct peaks of activity at the beginning of the light and dark period, akin to dawn and dusk in the natural environment, decreased activity around midday (siesta), and an extended period of inactivity during the night. In younger flies (3–5 days, as used in the SIN analysis) with either UAS-*hflDISC1* insert, there was lower average daytime activity compared to control flies (Figure 3A). This decreased activity primarily occurred after the siesta, from 15:00 until lights-off at 20:00 (Appendix A Figure A1A). In contrast, during the 12 h period of darkness (20:00–08:00), the locomotor activity remained the same in all genotypes (Figure 3B). Lower average daytime locomotor activity results corelate with smaller clustering coefficients of SIN analysis for both UAS-*hflDISC1* strains (Figure 2B,C). The lower locomotor activity of the UAS-*hflDISC1-3rd* flies could explain the lower frequency of interactions (clustering coefficient) but higher betweenness centrality where flies act as a bridge connecting different groups of flies (Figure 2D,E). The lower locomotor activity of UAS-*hflDISC1-2nd* flies consequently resulted in decreased connections (clustering coefficient), where they played a peripheral role with limited influence on, or access to, the rest of the network (lower betweenness centrality).

In aged flies (27–30 days old), we observed significant changes in activity during the day and night (Figure 3C,D). Both UAS-*hflDISC1* chromosome insertion lines were significantly less active than the controls at night, although only the UAS-*hflDISC1-2nd* flies were less so during the day (Figure A1B). These results confirmed our speculation that ageing might aggravate potential effects of the UAS-*hflDISC1* transgenic construct.

**Figure A2:**
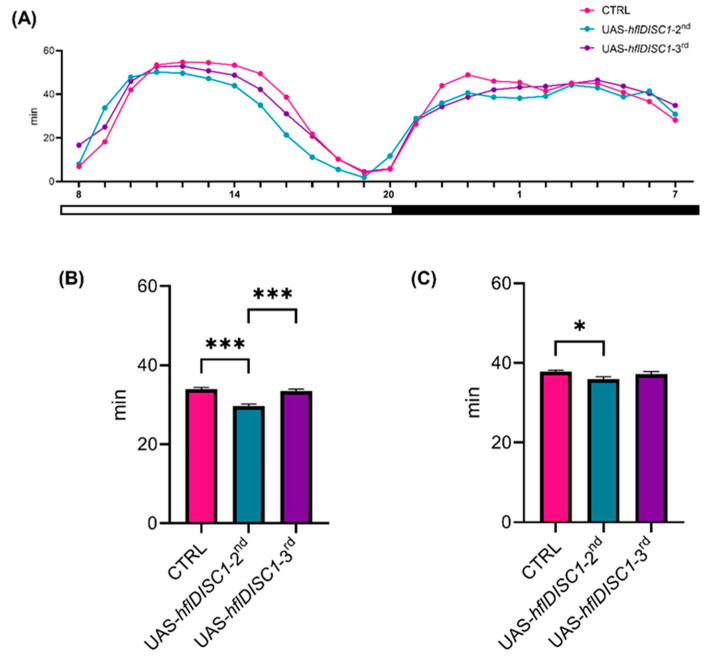

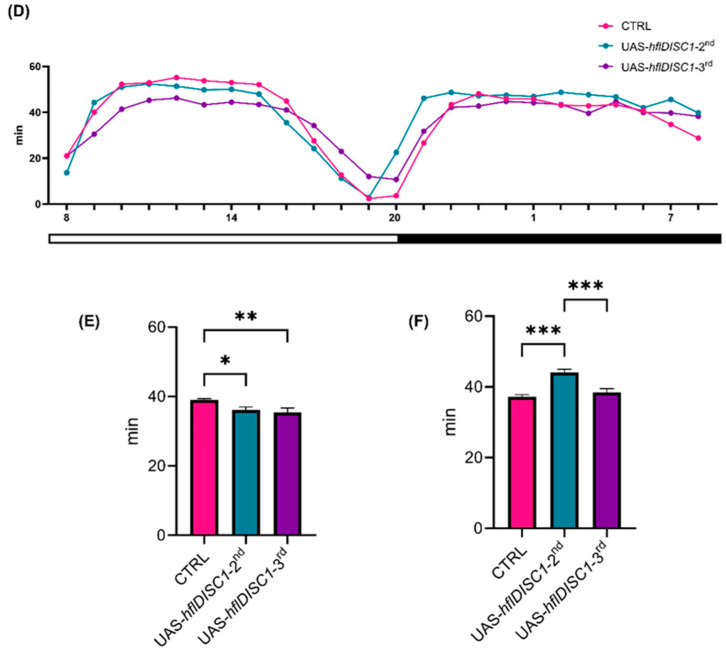
The change in sleep amount in UAS-*hflDISC1* transgenic flies is age-dependent. (**A**–**C**) young flies 3–5 days old, and (**D**–**F**) flies aged 27–30 days. Sleep was measured as the average value of the number of 5-minute inactivity periods from 16 flies per genotype. Data were then plotted as sleep per one hour (average of 60 min interval from 16 flies). Line graphs are an average of 5 consecutive days of sleep presented hour by hour (**A**,**D**). The bar graph represents day time sleep as average ± SEM recorded from 08.00 in the morning to 20.00 in the evening (**B**,**E**), while night sleep was average ± SEM from 20.00 in the evening to 08.00 in the morning (**C**,**F**). One-way ANOVA with Tukey’s multiple comparison post hoc test. *p* value style: APA < 0.05 (*), <0.01 (**), <0.001 (***).

**Figure A3:**
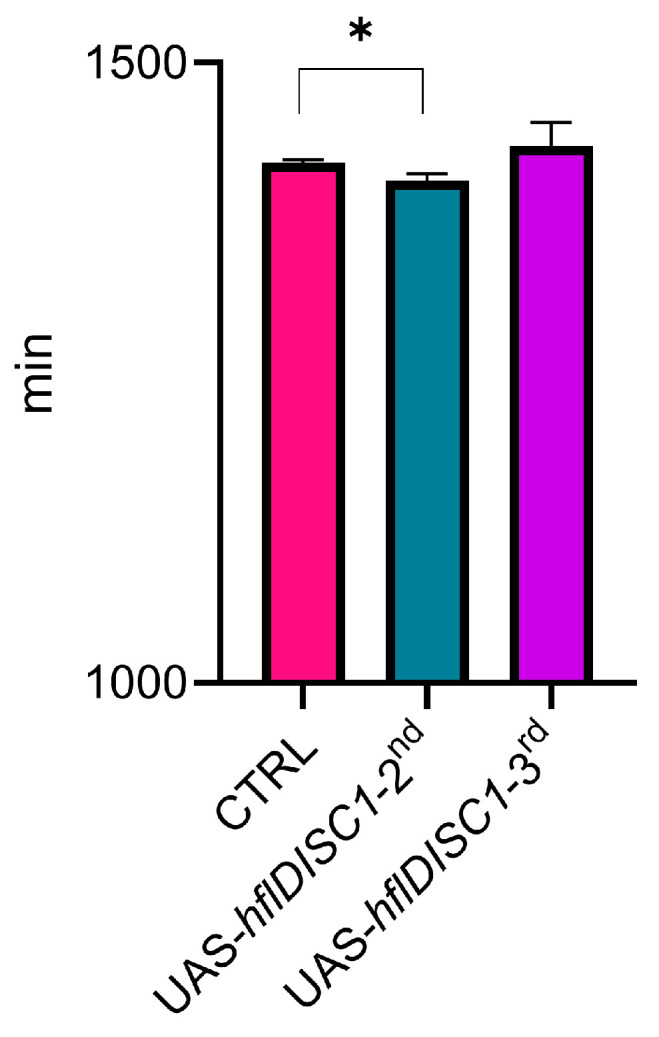
UAS**-***hflDISC1-2nd* transgenic flies have a decreased circadian period length. Activity was measured on flies 3–5 days old, under constant darkness for 5 days in row (*n* = 16 flies per genotype). The average value of the period length of individual flies was calculated from 5 days of activity recording using the ActoJ plugin in the ImageJ software. The bar graph presents the average ± SEM of period length. *p* value style: APA < 0.05 (*).

Sleep in flies is defined as a period of inactivity longer than 5 min, a cutoff that was empirically defined as the minimum time after which flies show the increased arousal thresholds [55,56]. Because sleep amount is derived from the locomotor activity, we reasoned that change in the amount of locomotor activity could influence the amount of sleep. Both UAS-*hflDISC1* insertion lines (second and third) displayed less daytime sleep with older flies showing a greater effect (Figure A2). The UAS-*hflDISC1*-*2nd* flies showed decreased sleep and lower locomotor activity during the day, indicating sluggishness when awake, but during the dark period, the locomotor activity decreased while the amount of sleep increased. Because sleep and activity are partially regulated by circadian factors, we examined the length of the circadian period for five days at 25 °C in constant darkness. Only the UAS-*hflDISC1*-*2nd* flies had a shortened length of the circadian period, suggesting altered circadian regulation (Figure A3).

### 3.3. UAS-hflDISC1 Insertion Affects the Climbing Ability

Considering that the UAS-*hflDISC1* construct impacted the locomotor activity of the transgenic flies, we evaluated their motor function and coordination by conducting the negative geotaxis test. In our assay, flies were placed in a tube, gently knocked to the bottom, and their ability to climb was quantified within a specified time (5 s). A group of ten flies was tested five times and five groups were tested for each genotype. This test is sensitive to the effects of ageing and neuronal degeneration, which results in a decreased negative geotaxis score.

**Figure 4:**
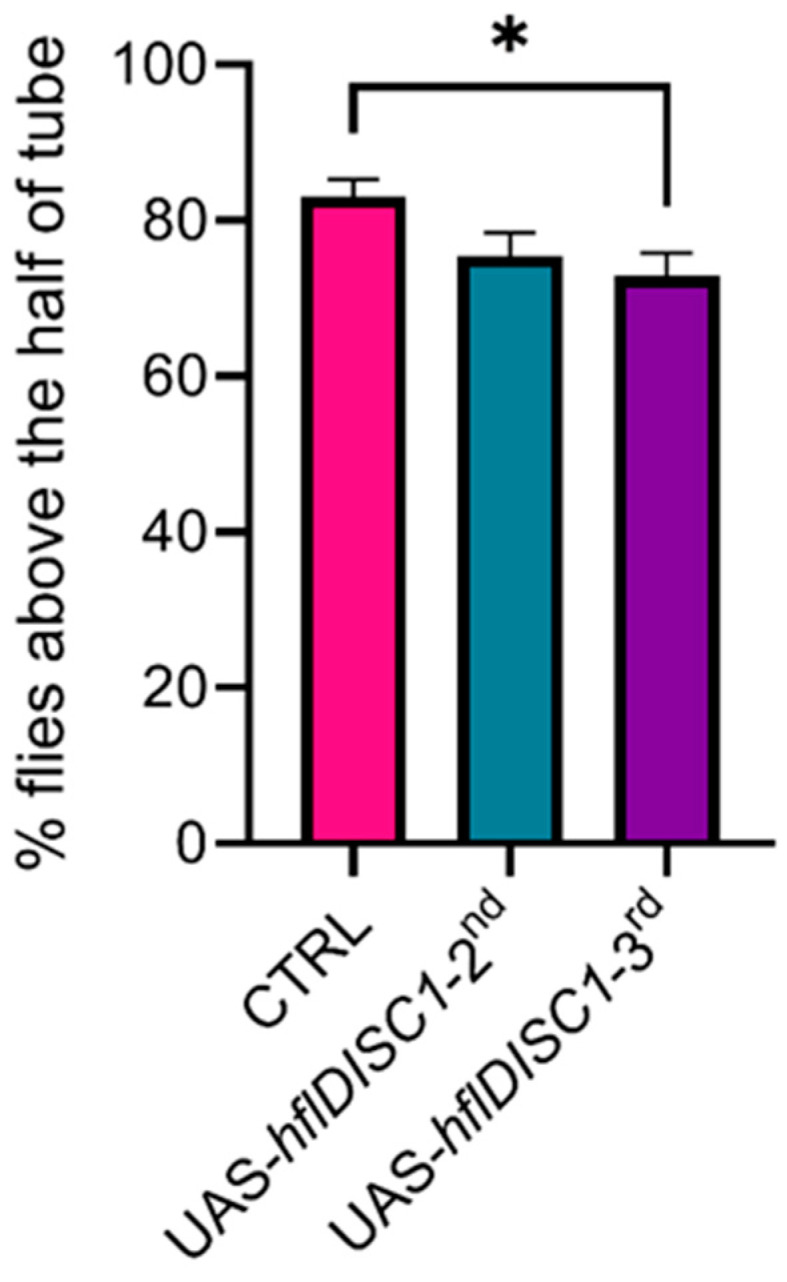
Decreased negative geotaxis in the UAS*-hflDISC1-3rd* transgenic flies. Test is performed on young flies 3–5 days old using driver lines UAS*-hflDISC1-2nd and* UAS*-hflDISC1*-3rd and *w^1118^* control. A total of 10 flies were placed in a tube without food 30 min for adaptation. The vials were then struck three times onto a surface for all the flies to fall to the bottom of the vial, and then they were photographed 5 s later, to determine the percentage of flies that climbed over a height of 5 cm. Measurements were repeated five times for each group, with an interval of one minute between measurements Each genotype was tested with five groups of ten flies, with each group undergoing five trials. The results are presented as the mean value of the measurements in triplicate ± SEM (*n* = 50 per treatment, performed in 5 replicas). One-way ANOVA test with Bonferroni correction, *p* value style: APA < 0.05 (*).

Only UAS-*hflDIS1*-3rd flies exhibited significantly lower climbing scores compared to the control group (Figure 4), which positively correlated with the reduced locomotor activity and changes in SIN parameters. The decrease in negative geotaxis suggests that the UAS-*hflDISC1-3rd* negatively impacts flies’ motor function, likely due to an effect on the motor neurons or potentially the functioning of the neurons in the central brain.

### 3.4. DISC1 Protein Is Expressed in the UAS-hflDISC1 Transgenic Flies

**Figure A4:**
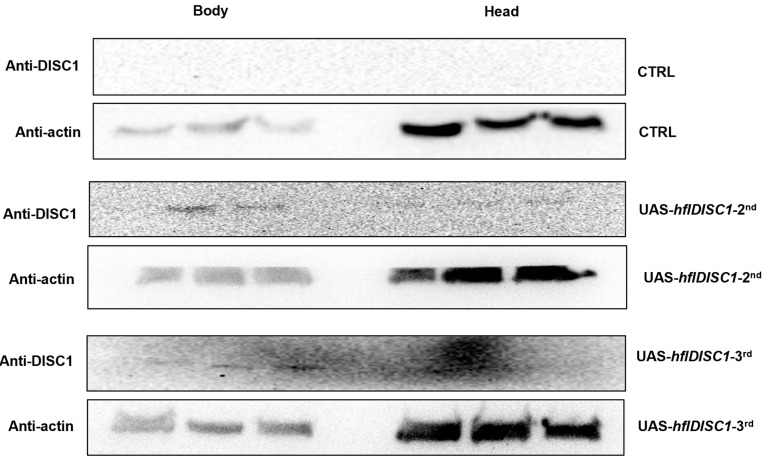
Expression of DISC1 protein measured in the body and head homogenates. Protein extracts were prepared form 3–5-day-old adult male flies in driver lines UAS*-hflDISC1-2nd and* UAS*-hflDISC1-3rd* and *w^1118^* control. Body samples were prepared from 5 bodies without heads, and head samples were prepared from 20 heads.

Due to various altered behavioral phenotypes seen in the UAS-*hflDISC1-2nd* and UAS-*hflDISC1-3rd* line, we reasoned that the source of the effects could be either the genomic insertion site or a leaky construct evident as background DISC1 expression in the absence of a GAL4 driver. An antibody against human DISC1 was used to investigate total proteins extracted from the homogenized heads and headless bodies to determine a potential presence in the brain and in the ventral nerve cord in the body, the equivalent of the spinal nerve cord in humans (Figure A4).

**Figure 5:**
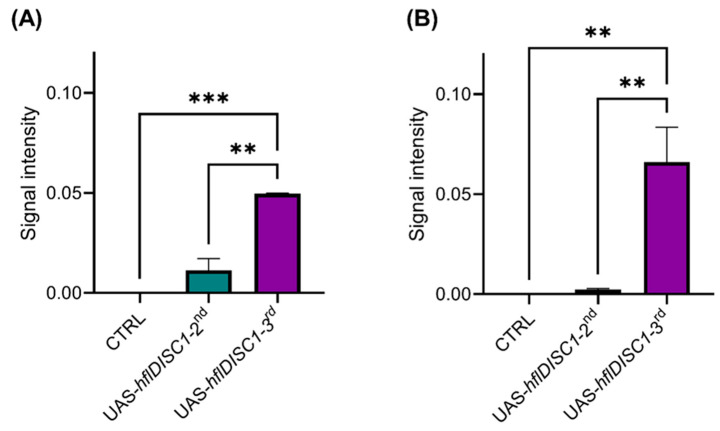
Expression of DISC1 protein measured in the body (**A**) and head (**B**) homogenates depends on insertion place**.** Protein extracts were prepared form 3–5-day-old adult male flies in driver lines UAS-*hflDISC1-2nd and* UAS-*hflDISC1-3rd* and *w^1118^* control. Body samples were prepared from 5 bodies without heads, and head samples were prepared from 20 heads. Data are presented as AVE ± SEM (*n* = 9). One-way ANOVA with Tukey’s multiple comparisons test. *p* value style: APA < 0.01 (**) and <0.001 (***).

The Western blot revealed that human DISC1 protein was detectable in both UAS-*hflDISC1* lines. The levels were notably higher in the UAS-*hflDISC1*-*3rd* flies compared to UAS-*hflDISC1*-*2nd*, in both heads and bodies (Figure 5A,B).

Interestingly, the 3rd chromosome contains more genes analogous to human genes that interact with *DISC1* compared to the insertion into the second chromosome, which harbors fewer such genes (Table A1). These results highlight the significant impact of chromosomal location on *hflDISC1* protein levels and its consequential behavioral effects in *Drosophila*.

### 3.5. UAS-hflDISC1 Leads to Redox Imbalance

Considering that both transgenic UAS*-hflDISC1* lines expressed the DISC1 protein, we wondered if the levels were sufficient to disrupt redox regulation. This is particularly important, as mental illnesses associated with DISC1 show oxidative stress phenotypes. Hydrogen peroxide (H_2_O_2_) is a reactive oxygen species (ROS) that when elevated can cause cellular damage, while glutathione (GSH) is a major antioxidant that helps neutralize ROS. The measurement of H_2_O_2_ levels reveals the extent of oxidative stress, while GSH levels indicate the capacity for antioxidant defense.

**Figure 6:**
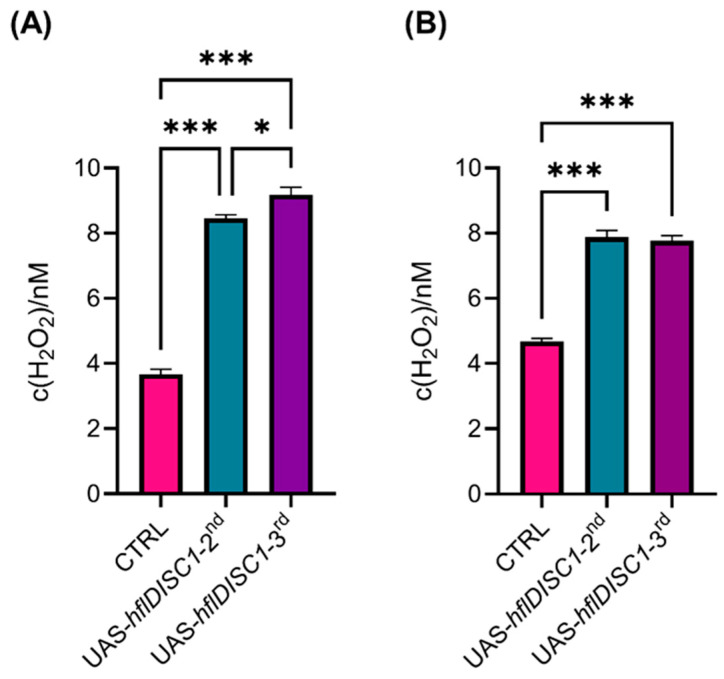
Increased H_2_O_2_ concentration in UAS-*hflDISC1* transgenic flies. Samples of 3–5-day-old UAS*-hflDISC1-2nd*, UAS*-hflDISC1-3rd*, and *w^1118^* control flies were collected from (**A**) 5 adult male headless bodies and (**B**) 32 adult male heads to prepare homogenates using dihydroethidium (DHE). All measurements were performed in triplicate (*n* = 9). Data are presented as AVE ± SEM. One-way ANOVA with Tukey’s multiple comparison post hoc test. *p* value style: APA <0.05 (*) and <0.001 (***).

Levels of H_2_O_2_ were significantly elevated in both the bodies and heads of transgenic UAS-*hflDISC1* flies compared to the control (Figure 6A,B). This elevation suggests an altered redox state, potentially influenced by *hflDISC1* expression. However, the two UAS-*hflDISC1* insertion lines did not differ in the head homogenate amount of H_2_O_2_ despite the significant difference in levels of DISC1 protein expression (Figure 5B and Figure 6B).

**Figure 7:**
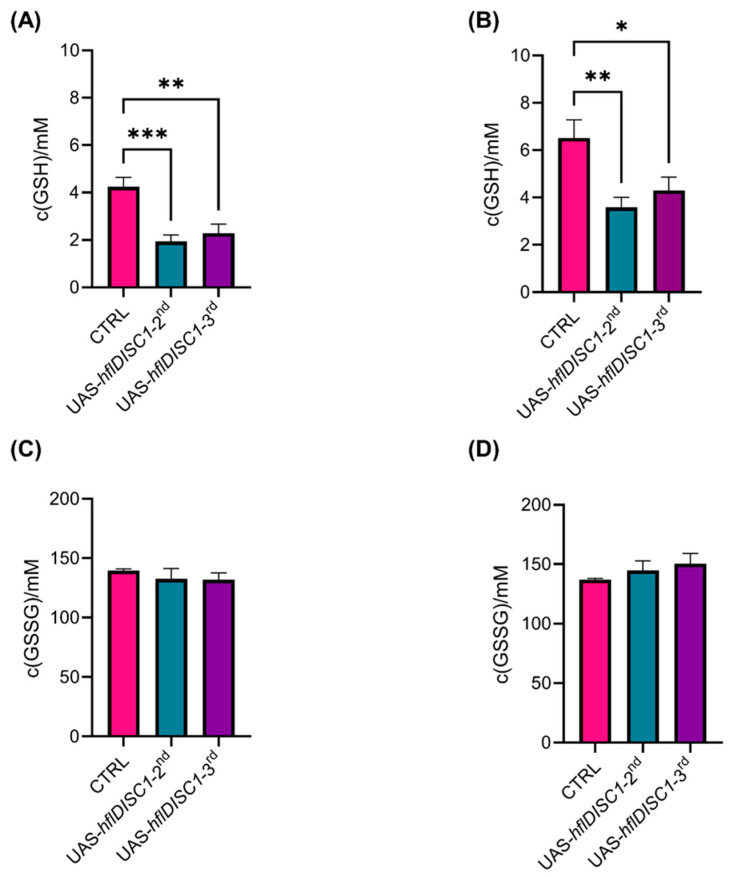
Decreased level of oxidized (GSH) but unchanged level of reduced glutathione (GSSG) in the UAS-*hflDISC1* headless bodies (**A**,**C**), and head homogenates (**B**,**D**). GSH (**A**,**B**) and GSSG (**C**,**D**) were measured using Ellman′s method reagent which reacts with the thiol group giving a product with the maximum absorbance at 415 nm. Using a calibration curve, GSH concentration was determined in homogenates of 5 adult headless male bodies or 32 adult male heads from 3–5-day-old adult UAS*-hflDISC1*-2nd, UAS*-hflDISC1-3rd*, and *w^1118^* control flies. All measurements were performed in triplicate (*n* = 9). Data are presented as AVE ± SEM. One-way ANOVA with Tukey’s multiple comparison post hoc test. *p* value style: APA < 0.05 (*), < 0.01 (**), <0.001 (***).

Cells have mechanisms to regulate and degrade H_2_O_2_ to prevent oxidative damage. Key antioxidant enzymes involved in this process include catalase, glutathione peroxidase, and peroxiredoxins, and their activities are influenced by the oxidative status within cells. To see if the change in H_2_O_2_ concentration is related to the activity of glutathione peroxidase, we measured the concentrations of both non-enzymatic glutathione (GSH), its active (reduced) form, and its oxidized form (GSSG). Levels of GSH were significantly lower in the headless bodies and the heads of UAS-*hflDISC1* lines (Figure 7A,B). The equal reduction in both transgenic lines correlates well with the similar increase in H_2_O_2_ and suggests a potential imbalance in cellular antioxidant defenses.

Interestingly, we did not detect significant differences in GSSG concentration between the UAS-*hflDISC1* lines and their respective control (Figure 7C,D). This indicates that while GSH levels are depleted, there is no corresponding increase in the oxidized form, suggesting a specific impact on the active antioxidant reserves rather than on the oxidative stress-induced oxidation of GSH.

## 4. Discussion

The *DISC1* (Disrupted in Schizophrenia 1) gene has attracted considerable attention for its potential impact on mental health and behavior. *DISC1* has been linked to a range of mental illnesses, including schizophrenia, bipolar disorder, and major depression [57]. Research shows that mutations or dysregulation of *DISC1* can affect neurodevelopmental processes, leading to changes in brain structure and function [58]. Various models, including genetic knockout mice and human cell models, have shown that aberrations in *DISC1* can result in behavioral changes and cognitive deficits, shedding light on the underlying mechanisms of mental illness and offering potential avenues for therapeutic intervention [59,60]. Furthermore, DISC1 is known to interact with other proteins of relevance to mental illnesses. An example is the interaction of DISC1 with dopamine D2 receptors [61,62], or the DISC1-Kalirin-7 interaction that highlighted the potential of inhibitors, such as FRAX486, in preventing synaptic deterioration and improving behavioral deficits in schizophrenia models [63].

Our goal was to study social interaction in flies expressing the *hflDISC1* construct in a cell-specific manner, and to this aim, we first created transgenic flies with human full-length DISC1 (*hfl*-*DISC1*) fused to the UAS promoter. This construct would enable future cell-specific brain expression, under the control of the GAL4 activator, to define the role that *hflDISC1* might have in the endophenotypes related to SCZ in humans. We have, however, discovered numerous behavioral and biochemical phenotypes in the two *UAS-hflDISC1* insertions on the second and third chromosomes. The numerous phenotypes observed in our study may result from the insertion site, but more likely they are due to the DISC1 expression, based on the evidence that the DISC1 protein is present in the bodies and heads of both transgenic strains and that some phenotypes are consistent between the two lines. 

There was no simple linear correlation between the amount of the DISC1 protein and the severity of the phenotypes, for which there are several potential explanations. Firstly, DISC1 is a complex scaffolding protein that affects and regulates many different cellular pathways. It is therefore likely that any effect would be far more complex than a linear relationship between protein expression and phenotype. Secondly, DISC1 is not ubiquitously and equally present in all neurons. For example, the UAS-*hflDISC1*-*2nd* line showed a lower expression of DISC1, relative to the UAS-*hflDISC1*-*3rd*, but nonetheless affected a significant number of phenotypes, which favors the explanation that the expression of DISC1 is present in selected neuronal groups. The altered amount of locomotor activity, sleep, and the circadian period length in the UAS-*hflDISC1*-*2nd* line all argue for potential DISC1 presence in neurons that regulate circadian behavior and sleep [64]. Considering that both sleep and the locomotor activity were more affected in older flies, relative to the controls, this supports the notion that the presence of DISC1 in those neurons leads to an age-related increase in the interreference with cellular functions. This could also be consistent with the buildup of DISC1 aggregates over time, causing a neurodegenerative-like effect [65].

The UAS-*hflDISC1*-*3rd* transgenic flies had a significantly higher expression of the DISC1 protein, relative to the UAS-*hflDISC1*-*2nd* line, but this did not correlate with the severity of the social interaction and locomotor activity phenotypes. However, UAS-*hflDISC1*-*3rd* flies had a decreased ability to climb vertical surfaces, which, together with the result showing increased DISC1 expression in the headless bodies, suggests that DISC1 could be present in the glutamatergic neurons. Glutamate is the excitatory neurotransmitter in the brain and, more importantly, the major excitatory neurotransmitter at the neuromuscular junction in invertebrates. Overexpression of DISC1 at the larval neuromuscular junction in *Drosophila* disrupts the formation of the glutamatergic synapses through the interaction with the fly homolog of Neurexin [38]. Thus, in UAS-*hflDISC1*-*3rd* flies, the expression of DISC1 at the neuromuscular junction, and potentially in the other central nervous system cells, could interfere with the speed and coordination of the climbing ability. An alternative, but less likely, explanation is that the site of insertion affected a wide range of neuronal groups with phenotype-specific functions.

DISC1 expression did not alter the SIN measure of global efficiency, indicating the formation of tightly knit groups. This shows that locomotor phenotypes of the two transgenic lines were not severe enough to interfere with this measure and that our recording setup (12 flies and 25 min of recording) allowed sufficient time for all the flies to come into contact with each other. However, at the local level, UAS-*hflDISC1* lines showed a lower clustering coefficient than controls, suggesting a lack of tight social clusters and indicating more diffuse and possibly random social interactions among individuals. The lower closeness centrality in the UAS-*hflDISC1*-*2nd* line indicates that the flies had less efficient access to all other flies in the network and that it takes longer for information or influence to reach another fly. This finding correlates with the lower locomotor activity such that it interferes with the frequency or duration of interactions with other flies. DISC1 presence in the UAS-*hflDISC1*-*3rd* line did not change closeness centrality but increased betweenness centrality, which suggests greater communication between different parts of the network. Since there is very limited knowledge about the role that different neurotransmitters play in the regulation of SIN parameters, it remains to be seen if the potential presence of DISC1 in the glutamatergic neurons interferes with specific SIN measures.

Our result showing the perturbed redox status in transgenic flies agrees with findings showing the involvement of DISC1 in mitochondrial trafficking [66] and the fact that a number of neurodegenerative and psychiatric diseases, including SCZ, are associated with increased levels of reactive oxidative species. Although the UAS-*hflDISC1*-*2nd* and UAS-*hflDISC1*-*3rd* lines each showed partially distinct behavioral phenotypes, they showed the same change in the redox parameters: elevated H_2_O_2_ concentration, lower GSH concentrations, and no differences from the control in the oxidized glutathione (GSSG). H_2_O_2_, a byproduct of metabolic processes, is tightly controlled because moderate-to-low concentrations are essential for cell signaling and for pathogen defense, while high concentrations of H_2_O_2_ are indicative of oxidative stress. Thus, the increased H_2_O_2_ and reduced GSH that we have observed is due to the increased oxidation of GSH to GSSG and likely impaired mitochondrial function caused by DISC1 expression. It has been established that hflDISC1 is present at the mitochondria [66,67], with deletion mutants of DISC1 leading to abnormal mitochondrial morphology [68,69] and distribution [21,70,71]. Elevated H_2_O_2_ levels in *hflDISC1*-expressing flies suggest disruptions in cellular redox signaling pathways, which could in turn affect neurotransmitter systems known to regulate behavior. In the future, it will be relevant to measure the concentration of different neurotransmitters in our UAS-*hflDISC1* lines and, even more relevant, in the DISC1 overexpressing lines.

Furthermore, aggregating DISC1 restricts mitochondrial movement [46,68,72]. Since *hflDISC1* forms aggregates in both transgenic rats and in subgroups of patients with mental illness, similar aggregates are likely to form in transgenic flies. It would therefore be interesting to compare the phenotypes of flies with the *hfl*DISC1 overexpression relative to the expression of a mutant forms of DISC1 [65], which would help define how DISC1 protein aggregates influence neuronal function.

## 5. Conclusions

To dissect the effects that the expression of the human full-length *DISC1* protein has on schizophrenia-related endophenotypes in flies, we created and characterized two novel UAS-*hflDISC1* transgenic fly lines, which would allow for neuron-specific DISC1 overexpression. Unexpectedly, UAS-*hflDISC1* transgenic lines showed the endogenous presence of DISC1 protein without transcriptional activation. DISC1 expression affected behavioral phenotypes, such as social interaction, locomotor activity and coordination, sleep and the regulation of the circadian periodicity, and resulted in perturbed redox balance. Some of these phenotypes could be explained by the sensitivity of behavior-specific neurons to DISC1 presence, although it needs to be determined if DISC1 is present ubiquitously or in a limited number of neurons. In the future, these transgenic lines can be used for neuron-specific overexpression of *hflDISC1* to examine the cellular effects in young and old flies.

## Data Availability

Raw data from the study can be found here https://repository.biotech.uniri.hr/islandora/object/biotechri:1015 accessed on 11 July 2024.

## Appendix A

### Appendix A.1. Altered Locomotor Activity and Sleep Patterns in the UAS-hflDISC1 Transgenic Flies

From the locomotor activity data, sleep was defined as a period of at least 5 min of inactivity (no infrared beam break) and expressed as the average minutes of sleep per hour [53].

Information on fly sleeping patterns can also be determined from these data, by looking at gaps in locomotor activity. Sleep is, therefore, to an extent, the opposite of locomotor activity (that is, when locomotor activity is low, sleep is high, and vice versa). However, because sleep and locomotor activity are regulated by a distinct neural mechanism, the inverse correlation is not always perfect. Furthermore, sleep in flies is defined in our assay as a period of inactivity longer than 5 min, a cutoff that was empirically defined as the minimum time after which flies show the increased arousal thresholds [64,73].

The changes in the amount of sleep seen during the day and night only partially correlate with the changes in the locomotor activity, supporting the distinct regulation of sleep and locomotor activity over 24 h. The UAS-*hflDISC1-2nd* line showed a decreased sleep amount during both the day and night, although the locomotor activity during the day was also lower (Figure A2B,C). This means that during the periods when they were awake, the UAS-*hflDISC1-2nd* flies were less active relative to the controls or the UAS-*hflDISC1-3rd* line. The UAS-*hflDISC1-*2nd flies slept less during and after the siesta time, similar to the decreased locomotor activity that was present at the same time of the day (Figure 3A). This shows that UAS-*hflDISC1-2nd* flies slept less but were also more sluggish when awake.

As with locomotor activity, there was a bigger effect on the amount of sleep in the older UAS-*hflDISC1* lines than the younger. Both the second and the third insertion lines slept less during the day (Figure A2E). In the case of the UAS-*hflDISC1*-2nd line, it again suggests a general sluggishness, because both the locomotor activity and sleep were low. During the night, the UAS-*hflDISC1-2nd* line shows increased amounts of sleep as would be intuitive based on the significantly decreased levels of locomotor activity (Figure A2D,F). These findings indicate that the insertion of UAS-*hflDISC1* on the second chromosome in *Drosophila* influences activity and sleep patterns, while the insertion on the third chromosome only affects daytime sleep in an age-dependent manner.

### Appendix A.2. Measurement of Free-Running Period under Constant Darkness

By recording activity for five days at 25 °C in constant darkness (DD conditions), we calculated the period length. This “free-running period” which refers to the natural period of the circadian rhythm in the absence of external cues is approximately 24 h and shows individual variation. Measurements of the period length from activity recorded in constant darkness were performed using the ActoJ plugin in ImageJ software https://imagej.net/ij/download.html (accessed on 20 April 2022.). Results represent the average period length for a minimum of 32 flies for each genotype.

### Appendix A.3. UAS-hflDISC1 Insertion Affects the Circadian Period Length

One of the major factors that regulate the timing of sleep and activity over 24 h is circadian genes. They are expressed in a small number of neurons in the fly brain but exert an effect on the entire physiology and behavior [74]. We reasoned that the differences in sleep and activity observed in the UAS-*hflDISC1* lines may be related to altered regulation of the circadian period length. We measured the period of the circadian locomotion, a common parameter that defines the circadian period in the free-running environment when flies are kept in constant darkness (DD). Because of the lack of environmental cues in constant darkness, this enables the organism to express the endogenous circadian period, which shows individual variation and is normally around, but not precisely, 24 h.

Our findings revealed that the insertion of *hflDISC1* on the second chromosome resulted in a shortened free-running period. This supports the notion that the locomotor and sleep phenotypes of the UAS-*hflDISC1-2nd* line might be related to a change in the length of the circadian period (Figure A3). These combined results underscore the multifaceted role of the *hflDISC1* insertion in modulating both activity and sleep patterns with implications for circadian disruptions in psychiatric conditions.

**Table A1:** A list of proteins expressed in *Drosophila* that are homologous to protein interaction partners of human DISC1, with respect to 2nd and 3rd chromosome localization in *Drosophila* [39,75].

Drosophila Homologs			Human DISC1 Interacting Proteins	
Protein Name	Protein Code	Localization (Chromosome)	Protein Name	UniprotKB
Phosphoglycerate kinase	CG3127	2L (2,746,880…2,753,068)	Phosphoglycerate kinase 1	P00558
Eukaryotic initiation factor 3 p40 subunit	CG9124	2L (5,051,193…5,053,094)	Eucaryotic translation initiation factor 3 subunit H	O15372
Sorting nexin 6	CG8282	2L (8,218,071…8,220,741)	Sorting nexin-6	Q9UNH7
Aminopeptidase P	CG6291	2L (16,908,229…16,910,418)	Xaa-Pro aminopeptidase 1	Q9NQW7
Dynamitin	CG8269	2R (8,891,832…8,893,339)	Dynactin subunit 2	Q13561
CG9003	CG9003	2R (11,641,477…11,651,616)	F-box only protein 41	Q8TF61
short stop	CG18076	2R (13,864,237…13,942,110)	Microtubule-actin cross_linking factor 1, isoforms 1/2/3/5	Q9UPN3
parcas	CG7761	2R (14,981,263…14,991,336)	SH3 domain-binding protein 5	O60239
Stretchin-Mlck	CG44162	2R (15,946,933…15,989,498)	A-kinase anchor protein 9	Q99996
Lissencephaly-1	CG8440	2R (16,178,826…16,185,103)	Platelet-activating factor acetylhydrolase IB subunit alpha	P43034
CG30291	CG30291	2R (21,007,755…21,009,576)	CDK5 regulatory subunit-associated protein 3	Q96JB5
Zipper sti	CG15792	2R (24,990,570…25,011,965)	Centrosomal protein of 63 kDa	Q96MT8
Cell division cycle 5 ortholog	CG6905	3L (357,851…361,383)	Cell division cycle 5-like protein	Q99459
trio	CG18214	3L (995,982…1,034,875)	Kalirin	O60229
misshapen	CG16973	3L (2,554,847…2,586,540)	TRAF2 and NCK-interacting protein kinase	Q9UKE5
Girdin	CG12734	3L (3,178,930…3,185,287)	Girdin	Q3V6T2
Exo70 ortholog	CG7127	3L (8,411,851…8,415,910)	Exocyst complex component 7	Q9UPT5
furry	CG32045	3L (9,631,834…9,679,103)	Protein furry homolog-like	O94915
nudE	CG8104	3L (9,899,241…9,903,031)	Nuclear distribution protein nudE-like 1	Q9GZM8
Mucin 68D	CG6004	3L (11,767,299…11,772,157)	Centrosomal protein of 170 kDa	Q5SW79
rogdi	CG7725	3L (17,049,512…17,055,454)	Protein rogdi homolog	Q9GZN7
Sec3 ortholog	CG3885	3L (17,421,787…17,425,217)	Exocyst complex component 1	Q9NV70
verthandi	CG17436	3L (27,136,525…27,157,999)	Double-strand-break repair protein rad21 homolog	O60216
Intraflagellar transport 54	CG3259	3R (14,302,428…14,304,458)	-	-
Dystrophin	CG34157	3R (19,461,085…19,597,288)	Dystrophin	P11532
dunce	CG32498	X (3,176,440…3,343,767)	cAMP-specific 3′,5′-cyclic phosphodiesterase 4B	Q07343
CG43689	CG43689	X (4,075,743…4,092,237)	-	-
dalao	CG7055	X (9,149,309…9,151,976)	SWI/SNF-related matrix-associated actin-dependent regulator of chromatin subfamily E member 1	Q969G3
β Spectrin	CG5870	X (17,657,372..17,669,371)	Spectrin beta chain, non-erythrocytic 1	Q01082
CG6867	CG6867	X (18,023,870..18,028,261)	Noelin	Q99784
